# Patient innovation under rare diseases and chronic needs

**DOI:** 10.1186/1750-1172-9-S1-O33

**Published:** 2014-11-11

**Authors:** Pedro Oliveira, Leid Zejnilovic, Helena Canhão, Eric von Hippel

**Affiliations:** 1 Católica-Lisbon School of Business and Economics, Lisbon, Portugal; 2 Carnegie Mellon University, Pittsburgh, PA, USA; 3 University of Lisbon School of Medicine, Lisbon, Portugal; 4 Massachusetts Institute of Technology, Cambridge, MA, USA

## Background

The patients afflicted by more than 7000 rare diseases are in “orphan” markets. They can expect little help from producers in the form of specialized products, and they have strong incentives to develop, or adopt solutions developed by peers, to help them cope with the diseases. We don’t know the extent to which patients and caregivers respond to these incentives and innovate, how they perceive impact of their solutions, and how they share their solutions with others.

The objectives of this work are: to measure frequency of patient innovation in a population of rare diseases patients; to measure efforts by patients to share their solutions with others; to explore which factors drive patients to come-up with solutions and share them with others.

## Material and methods

We developed a questionnaire comprised of 67 questions grounded in user innovation theory and adjusted to the rare diseases context. After validation, we applied the questionnaire over phone in a consecutive sample of 500 rare disease patients/caregivers. Subjects were selected from the list of individuals who contacted the helpline of an association of rare diseases patients from 2009 to 2012. The solutions reported by patients were validated for their novelty by two medical professionals. Additional data about diseases were collected from databases on rare diseases. We develop multivariate regression models to test relationships between our key variables and patient innovation and solution sharing.

## Results

263 (52.6%) of the respondents reported having a solution. 46 (9.2%) individuals reported solutions that they personally find valuable, and that are *also* evaluated as novel by expert medical evaluators. We find that the likelihood of patient innovation increases as education level increases, and with increase in perception of limitations imposed by the disease. 84 individuals shared their solutions, and the most common mode of sharing is patient-to-patient, reported by 74 individuals. There is a positive relationship between the impact of a solution on the respondents’ overall quality of life and likelihood of patients sharing their solutions, and inverted U relationship between age and the solution sharing.

## Conclusion

If anything like this fraction of innovators holds for the overall population of hundreds of millions of people world-wide estimated to be afflicted by rare diseases, patients and their caregivers who innovate to solve their own needs and improve their personal conditions may be a tremendous potential resource of information to improve management and care for many who are similarly afflicted.

